# Salivary testosterone and cortisol responses to seven weeks of practical blood flow restriction training in collegiate American football players

**DOI:** 10.3389/fphys.2024.1507445

**Published:** 2025-01-08

**Authors:** Paul E. Luebbers, Luke M. Kriley, Drake A. Eserhaut, Matthew J. Andre, Michael S. Butler, Andrew C. Fry

**Affiliations:** ^1^ John “Doc” Baxter Athletic Training and Human Performance Lab, Emporia State University, Emporia, KS, United States; ^2^ Jayhawk Athletic Performance Laboratory – Wu Tsai Human Performance Alliance, University of Kansas, Lawrence, KS, United States

**Keywords:** strength and power sport, athletes, resistance exercise, vascular occlusion, endocrinology

## Abstract

**Purpose:**

The purpose of this study was to examine the effects of a 7-week supplemental BFR training intervention on both acute and chronic alterations in salivary testosterone (sTes) and cortisol (sCort) in collegiate American football players.

**Methods:**

58 males were divided into 4 groups: 3 completed an upper- and lower-body split resistance training routine (H, H/S, H/S/R; H = Heavy, S = Supplemental, R = BFR), with H/S/R performing end-of-session practical BFR training, and H/S serving as the volume-matched non-BFR group. The final group (M/S/R) completed modified resistance training programming with the same practical BFR protocol as H/S/R. Athletes were further split into AM and PM training groups based upon their pre-determined training schedules, in cooperation with University strength and conditioning staff. Practical BFR consisted of end-of-session barbell bench press and back squat using 20% 1 repetition maximum (1RM) for 30-20-20-20 repetitions across 4 sets, with 45-seconds rest. Saliva samples were taken pre- and post- the first lower-body training sessions in week 1 and week 7 (i.e., test 1 and test 2) of the program, yielding four total. sTes and sCort were analyzed using 4-way (4 × 2 × 2 × 2) mixed model ANOVA’s.

**Results:**

Hormonal variables all exhibited main effects for time-of-day (p < 0.001). A significant group × time interaction effect (F_3,50_ = 3.246, p < 0.05) indicated increases in sTes post-training cycle for the H/S/R group only. Further, PM post-exercise sCort decreased from test 1 to test 2 (nmol·L^−1^: 95% CI: PM test 1 post-exercise = 10.7–17.1, PM test 2 post-exercise = 5.0–8.9). For the testosterone-to-cortisol ratio (T/C), AM pre-exercise was lower than PM (p < 0.05), with no change in post-exercise T/C for both AM and PM conditions when collapsed across testing times.

**Discussion:**

Overall, these findings suggest an ecologically valid method of BFR implementation is capable of inducing heightened concentrations of sTes in well-resistance trained American football athletes, providing additional insight on possible physiological mechanisms underpinning BFR’s ability to elicit beneficial muscle hypertrophy and maximal strength adaptations when performed during regimented training programs. Additionally, notable rises in T/C, and a null sCort response post-exercise were observed post-program for all groups, possibly indicative of positive physiological adaptation.

## Introduction

The addition of mechanically induced vascular occlusion, commonly referred to as blood flow restriction (BFR), during low-load resistance exercise has been shown to elicit muscle hypertrophy and even maximal strength adaptations comparable to that of traditional moderate-to-heavy load resistance exercise (Luebbers et al., 2014; Bjørnsen et al., 2019b; Reece et al., 2023; Thompson et al., 2024). In addition to changing local muscle oxygen saturation kinetics and microvascular blood volume acutely (Gray et al., 2023; Eserhaut et al., 2024), BFR resistance exercise may lead to longitudinal adaptations that are not easily attained with traditional training methods (Bjørnsen et al., 2019a). It is possible that supplementing traditional resistance training regimens with low-load BFR resistance exercise throughout the course of a full training cycle in well-trained athletes, in the form of an end-of-workout *finisher strategy* (i.e., supplemental BFR), may be an ecologically valid method for garnering these potentially additive physiological adaptations.

Research has demonstrated that resistance training with low-loads and high repetitions can result in significant increases in muscle size and strength, particularly when it is used in conjunction with BFR (Luebbers et al., 2014; Reece et al., 2023), with several proposed mechanisms for the positive muscular adaptations observed following the use of BFR training being put forth. For skeletal muscle hypertrophy outcomes, highly trained powerlifters have displayed improvements in quadriceps muscle cross-sectional area following a 7-week training block that included just 2-weeks of embedded lower body BFR training. Interestingly, preferential hypertrophy of type I muscle fibers was reported, suggesting that BFR may provide a novel stimulus for the highly metabolically active type I muscle fibers in strength and power athletes (Bjørnsen et al., 2019a). If so, BFR may be a training modality that offers supplemental benefit to normal resistance training regimens in individuals of this training status. Additionally, greater type I muscle fiber cross-sectional areas and denser intra-muscular capillary networks have been associated with improvements in oxidative metabolism, which may aid lifters with recovery between sets and across training sessions (Kacin and Strazar, 2011; Mitchell et al., 2018). Of specific interest are acute elevations in hormonal concentrations, as such increases signal the phosphorylation of various skeletal muscle cell receptors causing a variety of intracellular signaling cascades to take place, some of which directly influence gene expression and ribonucleic-acid (RNA) translation, both of which play regulatory physiological roles in skeletal muscle tissue growth and remodeling (Roberts MD et al., 2023). Acute endocrine responses during BFR resistance training have been measured in untrained and recreationally trained individuals in multiple instances, with elevations in growth hormone, testosterone, cortisol, norepinephrine, and salivary alpha-amylase being reported relative to non-BFR resistance exercise protocols (Iida et al., 2007; Madarame et al., 2010; Sharifi et al., 2020; Eserhaut et al., 2024). To date, no studies have examined the effects that practical BFR may have on the acute and chronic responses of the steroid hormones testosterone and cortisol when used in addition to a traditional high-intensity resistance training program performed by well-trained collegiate American football athletes.

Testosterone and cortisol have significant influence on anabolic and catabolic signaling cascades within skeletal muscle primarily via androgen and glucocorticoid receptors, respectively, and thus are of specific interest (Nicoll et al., 2019). Acute hormonal responses to resistance exercise are well documented with prolonged engagement in resistance exercise having previously been shown to elicit changes in resting hormonal concentrations (McMillan et al., 1993; Fry et al., 1998; Kraemer and Ratamess, 2005; Beaven et al., 2008; Walker et al., 2015). Decreases in post-exercise cortisol responses following a multi-week training regimen may be indicative of positive training induced adaptations and an improved physiological tolerance for a given resistance exercise prescription (Walker et al., 2015). Additionally, elevations in resting testosterone levels have been positively associated with greater countermovement jump heights in both male and female collegiate athletes, highlighting the partial influence of circulating testosterone levels on measures of lower body muscular power (Cardinale and Stone, 2006). Higher resting testosterone has also been correlated with greater knee extension (r = 0.88), and knee flexion (r = 0.84) maximal strength in a small cohort of middle-aged men (n = 7) (Baker et al., 2006). Further, positive correlations have been reported between the percent change in resting T/C ratio following a brief 3-week training program and competition totals (i.e., clean & jerk + snatch 1RMs) in elite competitive weightlifters, suggesting a favorable anabolic-catabolic hormonal balance at rest is indicative of favorable training adaptations in highly resistance-trained men (Fry et al., 2000). Mechanistically, the concentration of androgen receptors (AR) on skeletal muscle, through which testosterone initiates intracellular signaling cascades, has been shown to increase in men following prolonged engagement in regimented resistance exercise, with positive correlations observed between relative increases in AR concentrations and increases in mean muscle fiber cross sectional area (r = 0.62) (Ahtiainen et al., 2011). Collectively data appears to support the involvement of the androgen hormone testosterone in skeletal muscle structural adaptations and possibly neuromuscular performance improvements. Therefore, training modalities that may be capable of significantly amplifying the acute testosterone responses to resistance exercise, such as low-load BFR protocols, may provide strength and power athletes with additive training adaptations by way of hormonal alterations and other physiological effects when implemented regularly throughout moderate-to-long term training programs.

Thus, the purpose of this study was to examine the effects of a 7-week practical BFR training intervention on both acute and chronic changes in salivary measures of the steroid hormones testosterone (sTes) and cortisol (sCort) in collegiate American football players using a volume-matched design. It was hypothesized that the group performing the traditional high-intensity resistance exercise programming in combination with additional low-load practical BFR training at the end of their training sessions (H/S/R) would experience greater increases in post-exercise sTes concentrations both pre- and post- program than other groups, with H/S/R displaying significant decreases in post-exercise sCort concentrations following the training intervention in comparison to a volume-matched non-BFR group (H/S). Such findings would be indicative of significant BFR induced hormonal alterations, and general training adaptations resulting from progressively overloaded resistance exercise. Secondarily, it was also hypothesized that the cohort of athletes training in the AM would display higher sTes and sCort concentrations than the PM cohort, irrespective of group assignment, due to well-documented diurnal elevations in these hormones during the early morning hours (Weibel et al., 1995; van der Spoel et al., 2021).

## Materials and methods

### Experimental approach to the problem

The current investigation employed a pre-test post-test mixed model design (4 × 2 × 2 × 2) for a 7-week training intervention, with a total of 4 salivary samples for the analysis of sTes and sCort concentrations. Data presented are an analysis of hormonal data collected during a practical BFR resistance training study previously conducted by the authors and compliments the performance and limb circumference data previously published from this investigation (Luebbers et al., 2014).

### Subjects

Participants were recruited from a National Collegiate Athletic Association (NCAA) Division II American football team, from which 72 players volunteered to take part. All participants completed a seven-week, off-season football strength and conditioning program. Due to non-BFR related injuries or lack of compliance with the protocol, ten players were removed from the study yielding 62 players for baseline maximal strength testing. Athletes were then divided into four training groups (H, H/S, M/S/R, H/S/R; H = traditional high intensity strength and conditioning program, M = modified strength and conditioning program, S = supplemental 20% one repetition maximum lifting, R = practical blood flow restriction) (Luebbers et al., 2014). The group not performing traditional high-intensity lifting (M/S/R) was primarily comprised of skill position players (running backs, wide receivers, defensive backs, etc.) to ensure the heavily strength and power reliant positions (lineman and linebackers) were still exposed to high-intensity strength training during their off-season training. Participant characteristics and baseline maximal back squat and bench press strength data are provided in Table 1 (Luebbers et al., 2014).

**TABLE 1:** Participant characteristics.

Variables (units)	All (n = 62)	H/S/R (n = 17)	H/S (n = 14)	H (n = 15)	M/S/R (n = 16)
Age (yrs)	20.3 ± 1.1	20.6 ± 1.0	20.7 ± 1.3	20.0 ± 1.0	19.9 ± 1.2
Training experience (yrs)	7.1 ± 2.2	7.5 ± 2.8	7.9 ± 1.9	7.4 ± 1.2	5.5 ± 1.7
Body mass (kg)	99.1 ± 19.7	98.0 ± 18.3	107.5 ± 23.0	107.4 ± 19.8	85.2 ± 7.3
Back squat 1RM (kg)	190.0 ± 28.6	193.2 ± 25.0	196.6 ± 27.6	197.0 ± 35.1	174.4 ± 22.2
Bench press 1RM (kg)	127.1 ± 21.3	123.3 ± 20.7	135.1 ± 19.7	137.0 ± 24.4	115.2 ± 13.4

1RM, one repetition maximum; H, traditional high-intensity strength and conditioning program; S, supplemental 20% 1RM lifting protocol; R, practical blood flow restriction; M, modified strength and conditioning program.

Baseline 1RM strength data were previously published in (Luebbers et al., 2014) and are provided for descriptive purposes.

Permission to conduct the study was granted by the university’s Institutional Review Board. Details of the study were verbally explained to the entire team by the principal investigator at a team meeting. Each participant signed an informed consent document. All were deemed healthy and able to train by the university’s medical and athletic training staff.

### Procedures

#### Strength and conditioning program

The off-season training program consisted of four training sessions each week – two upper-body sessions and two lower-body sessions. Athletes were assigned by their coaches to attend one of three training sessions each day for the entirety of the 7-week program, held at either 7:15 a.m. (AM condition), or the afternoon times of 1:30 p.m., and 3:30 p.m (combined for PM condition) to accommodate subjects’ class schedules. Of the four groups, H/S/R performed the supplemental 20% 1RM lifting protocols under conditions of practical BFR, with the H/S group performing the same traditional high-intensity training paired with supplemental lifting protocols, but without practical BFR and therefore serving as a volume-matched non-BFR comparative condition. The H group performed only the traditional high-intensity resistance training, with the final M/S/R group completing a modified strength training program with the same practical BFR protocol as H/S/R, with the only difference being M/S/R did not perform back squats, lateral squats, bench press, or lockout press. Thus the M/S/R group allows for the assessment of whether or not a resistance training session performed with end-of-session practical BFR that does not contain traditional high-intensity exercises elicits comparable steroid hormone responses to the three other comparative high-intensity groups (H/S/R, H/S, H). Lastly, subjects were instructed to avoid performing any non-program related resistance training, with the exception of football practice related activities, for the duration of the study. Detailed descriptions of representative upper- and lower-body training sessions for all four groups are provided in Table 2.

**TABLE 2:** Representative training sessions for each group: total volume-load^a^.

Lower-body session								
Exercise	Set	Rep	Load (kg)	%1RM	H/S/R Vol-load	H/S Vol-load	H Vol-load	M/S/R Vol-load
Squat	1	8	130	65%	1,040	1,040	1,040	—
	1	6	140	70%	840	840	840	—
	1	4	160	80%	640	640	640	—
	1	2	170	85%	340	340	340	—
	1	2	180	90%	360	360	360	—
Lateral squats	3	5	130	65%	1,950	1,950	1,950	—
Good mornings	3	6	45.5	—	819	819	819	819
DB lunges^b^	3	8	22.7	—	544.8	544.8	544.8	544.8
Glute-Ham raises	3	6	11.4	—	205.2	205.2	205.2	205.2
Supplemental squats	1	30	40	20%	1,200	1,200	—	1,200
	3	20	40	20%	2,400	2,400	—	2,400
Total vol-load (kg)					10,339	10,339	6,739	5,169

Upper-body session								
Bench press	1	8	81	65%	650	650	650	—
	1	6	87.5	70%	525	525	525	—
	1	4	100	80%	400	400	400	—
	1	2	106.25	85%	215	215	215	—
	1	2	112.5	90%	225	225	225	—
Lockout press	4	2	81	65%	650	650	650	—
Upright rows	3	6	45	—	810	810	810	810
Single-arm DB rows^b^	3	8	45.5	—	1,092	1,092	1,092	1,092
Triceps DB extension	3	10	25	—	750	750	750	750
DB Hammer Curls^b^	3	10	22.7	—	681	681	681	681
Supplemental bench	1	30	25	20%	750	750	—	750
	3	20	25	20%	1,500	1,500	—	1,500
Total vol-load (kg)					8,248	8,248	5,998	5,583

H/S/R and M/S/R groups performed supplemental squat and bench exercises with practical blood flow restriction.

Training session tables are adapted from (Luebbers et al., 2014).

^a^ Volume-load calculations are for an example participant with a 200 kg back squat one-repetition maximum (1RM) and a 125 kg bench press 1RM.

^b^ DB, dumbbell; H, traditional high-intensity training program; S, supplemental 20% 1RM lifting protocol; R, practical blood flow restriction; 1RM, one repetition maximum.

#### Practical blood flow restriction

Practical BFR training consisted of end-of-session barbell bench press and back squat (performed on each of their respective training days) using 20% 1 repetition maximum (1RM) for 30-20-20-20 repetitions across 4 sets, with 45-second inter-set rest intervals. These end-of-session exercises are denoted as “S” in the groups H/S/R, H/S, and M/S/R. The method of practical BFR application involved the use of powerlifting elastic knee wraps with a hook-and-loop closure (Grizzly Fitness, Kitchener, Ontario, Canada). Knee wrap dimensions were 7.6 × 167.6 cm (3.0 × 66.0in). For the BFR bench press, the wraps were applied to the proximal-most end of the upper extremities above the biceps and below the deltoid. Likewise, for BFR back squats wraps were applied to the proximal-most end of the lower extremities at the top of the thigh near the inguinal crease. The BFR wraps were initially applied bilaterally with very light tension, just secure enough to remain in place on both limbs. Before initiating the light-weight supplemental bench press and back squat protocols, the wraps were then pulled to 7.6 cm (3.0in) of overlap (relative to the lightly secured pre-exercise state) as measured by graduated 1.3 cm silver markings and worn continuously across the 4 sets of 30-20-20-20 repetitions with 45-seconds of inter-set rest. Wraps were then removed immediately following set 4 (Luebbers et al., 2014). The two groups that performed the supplemental end-of-session bench press and back squats, denoted as “S,” with the addition of practical BFR, denoted as “R,” are H/S/R and M/S/R.

#### Saliva sample acquisition and analysis

Saliva collection is a non-invasive and time-efficient method often used for the collection of analytes in field-based settings and has greater ecological validity in comparison to blood draws. Additionally, previous research documents the strong association between salivary free testosterone and total serum testosterone concentrations (Lane and Hackney, 2015), along with salivary free cortisol and serum total cortisol concentrations (Dorn et al., 2007), both at rest as well as following moderate and high-intensity exercise. During the training program, four subjects failed to provide complete sets of salivary samples, and thus samples from the 58 remaining participants were used for analysis. Four total saliva samples were collected in total from each participant. The first set of two samples was collected on the first training session of week 1 (a lower-body/squat workout): just prior to the workout (Pre-Ex, Test 1a) and again upon conclusion of the workout (Post-Ex, Test 1b). The final set of two samples were collected during session 1 of week 7 (a lower-body/squat workout): just prior to the workout (Pre-Ex, Test 2a) and again upon conclusion of the workout (Post-Ex, Test 2b). These sampling times were chosen as each training session was the first of their respective weeks, preceded by 2 days of complete rest over the weekend offering a form of built in control for any carry-over fatigue effects that may have occurred had a training session been performed on the days immediately prior to saliva sample collection. Thus, pre- and post-program hormonal concentrations reflect the effects of 6-weeks plus 1-day of the planned strength and conditioning program, despite athletes performing 3 additional training days in week 7 to conclude their pre-planned phase of training. On the 2 days of sample collection, participants were asked to abstain from eating or drinking for at least 1 h prior to the resting sample collections (Pre-Ex, Test 1a & Pre-Ex, Test 2a). Upon arriving at the locker room, participants sat and rested for approximately 5 min. Saliva samples were then obtained using Salimetrics Oral Swabs (Salimetrics, State College, PA). Participants then proceeded to the weight room for their workouts. Upon completion, participants returned to the locker room and rested for approximately 5 min. Saliva samples were again collected at the conclusion of that time period (Post-Ex, Test 1a and Post-Ex, Test 2b). Saliva samples were put on ice immediately upon collection and taken to a freezer where they remained at −80°C for the duration of the study. For analysis, all saliva samples were removed from the freezer, packed in dry ice and shipped overnight to the Salimetrics laboratory facility in State College, PA. All samples were assayed in duplicate for sTes using a high-sensitivity enzyme immunoassay (EIA) with a lower limit sensitivity of 5.20 pmol/L. Samples were also assayed in duplicate for sCort using EIA with a lower limit of sensitivity of <0.19 nmol/L. Mean intra- and inter-assay coefficients of variation were less than 10% for both hormones.

### Statistical analyses

Hormonal variables were analyzed using 4-way (4 × 2 × 2 × 2) mixed model ANOVAs based on the four groups (H/S/R, M/S/R, H/S, H), two test times (test 1 and test 2; i.e., pre and post study), and two training times (pre-exercise and post-exercise), and two times-of-day (AM and PM). Statistical design included between subjects (group, time of day, group x time of day) and within subjects (test time, training time, test time x training time). Assumptions of sphericity were supported by non-significant results using Mauchly’s test of sphericity for all comparisons (p > 0.05), while normality of data was verified with the Shapiro-Wilk test (p > 0.05). Post hoc analyses for pair-wise comparisons were performed with 95% confidence intervals using Bonferroni adjustments for multiple comparisons. Significance was set *a priori* at p < 0.05, and all data are reported as mean ± SD.

## Results

**FIGURE 1:**
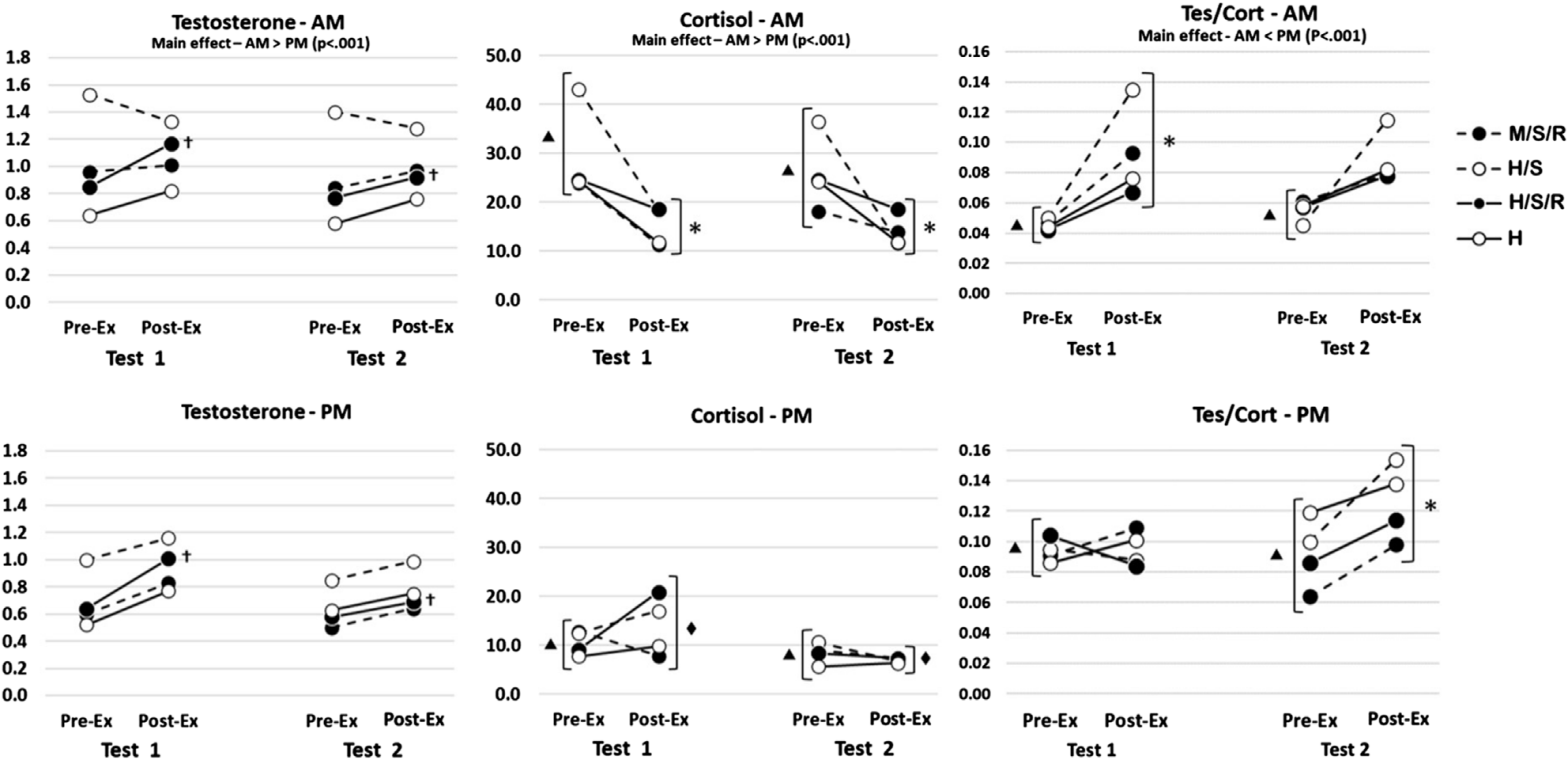
Graphical display of mean hormonal concentrations for all training groups throughout the 7-week training intervention. H/S/R, traditional high-intensity training with supplemental practical BFR; H/S, traditional high-intensity training with volume-matched non-BFR supplemental exercise; H, traditional high-intensity training only; M/S/R, modified training program with supplemental practical BFR. (^†^) H/S/R sig. greater than Pre-Exercise (p < 0.05); (*) All groups, sig. difference between Pre- and Post-Exercise (p < 0.05); (^▴^) All groups, AM sig. different from PM (p < 0.05); (^◆^) All groups, Test 2 sig. > Test 1 (p < 0.05).

As expected, due to diurnal variations, all hormonal variables exhibited significant main effects for time-of-day (cortisol, AM>PM, df = 1,50, F = 37.791, p < 0.001; testosterone, AM > PM, df = 1,50, F = 18.758, p < 0.001; T/C, AM < PM, df = 1,50, F = 13.890, p < 0.001). As such, besides comparing the responses to each of the four conditions (H/S/R, M/S/R, H/S, H), differences in the AM and PM responses will be noted. Results for saliva-derived steroid hormone concentrations are illustrated in Figure 1.

For sTes, the 4-way interaction was not significant (df = 3,50; F = 0.023; p = 0.995). The only significant interaction was for group × training (df = 3,50, F = 3.246, p < 0.05), indicating that only the H/S/R group exhibited significant increases following all training sessions. Although not significantly different, this appeared to be driven primarily by the acute response at test 1.

For cortisol, the 4-way interaction was not significant (df = 3,50; F = 0.498; p = 0.685). The 3-way interaction between time-of-day × test × pre/post-exercise was significant (df = 1,50; F = 4.149; p = 0.047). Post hoc analyses indicated the AM pre-exercise sCort concentrations were greater than the PM pre-exercise concentrations (nmol·L^−1^; 95% CI; AM test 1 pre-exercise = 22.5–35.5, AM test 2 pre-exercise = 20.0–27.2, PM test 1 pre-exercise = 5.5–15.5, PM test 2 pre-exercise = 5.6–11.2), and the pre- to post-exercise concentrations decreased for the AM tests (AM test 1 post-exercise = 10.9–19.1, AM test 2 post-exercise = 10.8–15.9) but not the PM tests (PM test 1 post-exercise = 10.7–17.1, PM test 2 post-exercise = 5.0–8.9). These results also indicate the PM post-exercise sCort concentrations decreased from test 1 to test 2, which was not observed for the AM training sessions.

When both the sTes and sCort concentrations were used to determine the T/C ratio, the 4-way interaction was not significant (df = 3,50; F = 0.358; p = 0.783). The 3-way interaction for T/C between time-of-day × test × pre/post-exercise was significant (df = 1,50; F = 4.732; p = 0.034). Post hoc analyses indicated the AM pre-exercise T/C ratios were lower than the PM pre-exercise concentrations (ratio; 95% CI; AM test 1 pre-exercise = 0.023–0.068, AM test 2 pre-exercise = 0.037–0.073, PM test 1 pre-exercise = 0.077–0.111, PM test 2 pre-exercise = 0.078–0.106), and the pre- to post-exercise ratios increased for the AM tests at test 1 but not test 2 (AM test 1 post-exercise = 0.069–0.116, AM test 2 post-exercise = 0.067–0.110), while the T/C ratio for the PM tests did not change for test 1 pre- to post-exercise, but increased for PM test 2 (PM test 1 post-exercise = 0.077–0.113, PM test 2 post-exercise = 0.109–0.143). These results also indicate that the T/C post-exercise ratios for both AM and PM conditions did not change from test 1 to test 2.

## Discussion

The principle findings of this investigation are as follows: First, the group performing traditional high-intensity resistance training with the supplementation of low-load practical BFR training (H/S/R) experienced a significantly elevated post-exercise sTes response at Test 1, with rises in sTes for the other 3 groups failing to reach statistical significance. Second, while not statistically significant, the H/S/R group displayed greater absolute post-exercise sCort levels at Test 1 than the other groups. This suggests exercising with vascular occlusion may lead to greater sCort levels than performing the same volume of work without BFR (H/S/R > H/S), which would be in agreement with prior reports (Eslami et al., 2019; Eserhaut et al., 2024). Importantly, the M/S/R group, which also supplemented their modified resistance training regimen with practical BFR training saw no significant changes in sCort post-exercise at Test 1, suggesting that high-intensity back squats performed before practical BFR may have latent additive effects on circulating hormonal concentrations. Lastly, post-exercise sCort concentrations for all groups displayed significant reductions at Test 2, indicative of an enhanced physiological tolerance to the resistance exercise stressors of the program and beneficial performance adaptations, which aligns with the improvements in maximal back squat and bench press strength observed with these athletes (Luebbers et al., 2014).

Despite no significant interaction effects warranting targeted post-hoc comparisons for H/S/R, PM, Test 1 sCort Pre- and Post-Ex (nmol/L; Pre-Ex: Mean = 9.070, 95% CI [-0.394–18.534]; Post-Ex: Mean = 20.890, 95% CI [14.902–26.878]), the Post-Ex mean was over double the Pre-Ex mean (2.3-fold increase). While this is not a conventional statistically significant change, some degree of increase may have still occurred. Post-exercise sCort concentrations of 20 nmol/L or above can be considered fairly robust following resistance exercise, as in Eserhaut et al. (2024) mean post-exercise sCort was ∼16 nmol/L following a momentary task failure, low-load (30% 1RM) bilateral seated leg extension protocol performed with blood flow restriction in the afternoon-to-evening in highly-resistance trained men. Similarly, Crewther et al. (2008) show peak sCort responses of ∼17 nmol/L after high volume (10 sets) and moderate-to-high load (75% 1RM) machine squat resistance training bout performed in the afternoon-to-evening time of day. These reports may support the trend toward significant elevations in sCort post-workout for the H/S/R PM cohort at Test 1. Additionally, the sCort responses in this study occurred following an ecologically valid strength and conditioning program which used practical BFR as a “supplement,” and therefore suggests the use of BFR as an end-of-session *finisher strategy* may still elicit significant increases in sCort when performed by highly resistance-trained men. Further studies measuring sCort responses following a replication of the heavy-load training followed by practical BFR with comparably well-resistance trained athletes are needed to better elucidate the significance of the sCort responses observed.

The androgenic analyte sTes expressed statistically significant increases post-training in the H/S/R group, with the volume-matched non-BFR group (H/S) failing to induce such changes. This suggests that the addition of limb occlusion via practical BFR to a given volume of resistance exercise may also amplify androgen concentrations in highly trained men. To date, findings on BFR’s ability to elevate testosterone are mixed with studies showing no post-exercise elevation following upper body BFR training (Reeves et al., 2006), and significant increases following one set of 30 repetitions followed by two sets to failure for both seated leg extensions and leg curls (Madarame et al., 2010). More recent work reports no significant elevation in serum testosterone following a fairly conservative 20%1RM × 4 sets × 15 repetition BFR protocol (Laurentino et al., 2022). Importantly, the aforementioned studies all recruited recreationally active men, and thus, given documented differences in testosterone responses to resistance exercise in well resistance trained men relative to untrained (Sontag, 2019), and endurance trained cohorts (Kraemer et al., 1989), it is speculated that lower training statuses paired with the use of relatively conservative BFR training protocols in some studies may explain the lack of post-exercise testosterone elevations in previous findings.

The significant post-exercise elevation in sTes for H/S/R may be due to a number of effects that the continuous application of lower-body BFR across multiple sets of resistance exercise has on the acute exercising physiology of trained-men. In comparison to a volume-matched non-BFR leg extension protocol, continuous BFR markedly blunts the rate at which oxygen saturated blood reperfuses into the microcapillaries of exercising musculature during inter-set rest intervals, likely contributing to impaired local recovery at the level of the muscle during multi-set training protocols (Eserhaut et al., 2024). With fixed-repetition BFR protocols such as the 20% 1RM x 4 sets x 30-20-20-20 regimen used in this study, continuous BFR undoubtedly leads to athletes terminating sets 2, 3, and 4 in relatively close proximity to momentary task failure (on average), requiring greater levels of exertion across a given protocol as local muscle fatigue manifests (Sieljacks et al., 2019). A combination of impaired local muscle tissue oxygen reperfusion and closed proximities to momentary task failure likely contributed to the statistically significant BFR-induced acute increases in sTes, observed for the H/S/R group training in the PM (Post-Ex, Test 1). BFR’s ability to make fairly mundane light loads (e.g., 20%–30% 1RM) significantly more challenging, paired with the performance of fairly high repetitions with abbreviated rest periods (30–60 s) likely generating notable metabolic demand, a proposed contributing mechanism to resistance exercise induced testosterone elevations (Vingren et al., 2010).

While the H/S/R group experienced significant post-exercise sTes elevations at Test 1 (PM), and non-significant increases in mean sCort, the group performing the same end-of-session practical BFR protocol in the absence of any heavy-intensity resistance exercise preceding it failed to display statistically significant increases in sTes, with a non-significant reduction in mean sCort Post-Ex. The latent effects of robust testosterone and cortisol concentrations caused by the high-intensity back squats that preceded sample collection in the H/S/R group cannot be dismissed when interpreting these data, as heavy load compound exercises using large volumes of skeletal muscle mass are known to induce marked elevations in the hormones of interest (Kraemer et al., 1989; Kraemer et al., 1999; Ratamess et al., 2005; Rønnestad et al., 2011). Further, while not statistically significant, absolute mean sCort values were greater Post-Ex for all three groups performing heavy-load training (barbell squats and lateral squats) at Test 1 (PM). Peak sCort concentrations are known to occur 15–45 min following the onset of a stressor (Dickerson and Kemeny, 2004) with peak sTes manifesting on a comparable timeline depending upon the exercise protocol performed (Crewther et al., 2008; Beaven et al., 2010). Thus, residual effects from the traditional higher intensity resistance training performed early in the training session may have partially contributed to the post-exercise sTes and sCort concentrations measured in the H/S/R group. In isolation, lower-body continuous BFR protocols significantly elevate sCort in highly resistance trained men (Eserhaut et al., 2024), but it remains possible that performing high-intensity compound exercises prior to low-load BFR may permit larger post-exercise sTes and sCort concentrations to be obtained. Whether this exercise sequencing has an additive effect on longitudinal muscle growth and/or strength and power adaptations is unknown and beyond the scope of this investigation. It is speculated that performing traditional moderate-to-heavy load compound exercises prior to low-load practical BFR would be more beneficial throughout a moderate-to-long term training program, potentially due to the summative effects of larger acute hormonal concentrations, but even more so when prioritizing the development of the strength and power adaptations pertinent to success in a sport such as American football, where exercises performed first in a training session in the presence of minimal fatigue appear to see greater improvements in maximal strength and power in comparison to those performed later (Nunes et al., 2021). Thus, positioning BFR training protocols at the end of strength and conditioning sessions is likely best practice when designing programs for strength and power athletes that seek to implement this training method.

When evaluating sTes and sCort as a ratio (T/C), all groups in the AM cohort expressed significant elevations in T/C post-exercise at test 1 and test 2 due to sCort’s pronounced fall from its diurnal apex in the early morning hours. Of interest however, is the significant post-exercise elevation in T/C for the PM cohort at test 2, as such a rise indicates sTes levels still increased to an extent at the end of the 7-week training program while sCort concentrations remained unchanged (see Figure 1). This bolsters the aforementioned finding of null sCort responses post-exercise potentially being indicative of athletes adapting to the demands of training over time, and as a result yielding lower levels of the commonly studied stress hormone following resistance exercise. Indeed, a greater T/C ratio has been associated with greater lower body power (Luebbers et al., 2022), and improvements in other performance outcomes in a number of other reports (Fry et al., 2000; Ahtiainen et al., 2003; Crewther et al., 2020), possibly related to athletes possessing a favorable *anabolic-to-catabolic* balance, and heightened state of psycho-physiological readiness (Cook and Beaven, 2013). Thus, it is likely that positive physiological adaptations occurred alongside the improvements in maximal back squat and bench press strength performances in this cohort of American football players, across all program groups (H/S/R, H/S, H, and M/S/R) (Luebbers et al., 2014).

With respect to time-of-day differences, athletes lifting in the AM experienced greater absolute pre- and post-exercise sTes and sCort levels than PM, which corroborates the long-running history of the known diurnal rise in these hormones during the early morning hours after awakening (Weibel et al., 1995; van der Spoel et al., 2021). Waking up in the morning is associated greater concentrations of serum cortisol, with concentrations reaching an apex 30–60 min after awaking (Hellhammer et al., 2007). This morning rise in cortisol accelerates the activity of select metabolic pathways (Florini, 1987), with resultant increases in skeletal muscle protein turnover (Brillon et al., 1995). It is suggested that the concomitant rise in testosterone, which typically occurs 30–60 min following cortisol’s diurnal elevation, may serve to counter-act cortisol’s protein degradation effects in a form of physiological checks and balances system (Kraemer, 1988). After these natural rises, the fall of plasma cortisol in the later morning hours can blunt the magnitude of exercise-induced increases. Interestingly, H/S/R was the only group to display significant post-exercise elevations in sTes in the AM cohort, suggestive of a rather robust exercise induced elevation. This is not indicative of AM training being more, or less, effective than PM but rather that the diurnal effects on circulating hormonal concentrations are rather marked, highlighting the importance of sampling sTes and sCort in the afternoon and evening as an often-preferable study design decision when assessing the effects of exercise on exercise-induced alterations in endocrine physiology. Further, little-to-no difference in muscle strength and/or hypertrophy has been reported in other studies when groups perform AM versus PM training (Grgic et al., 2019), with the effectiveness of routine early morning versus late evening workouts being partly related to an individual’s personal preference (Blazer et al., 2021).

Lastly, given the applied nature of this investigation, there are important limitations to consider. The use of elastic knee wraps in a practical manner to induce blood flow restriction does not allow for the precise measurement of pressure applied to the extremities, and thus no individualized percentages of arterial occlusion pressure (AOP) could be prescribed. However, Wilson et al. (2013) report that elastic wraps of the same width as those employed in this study (7.6in wide) are capable of impeding the venous outflow of blood, along with partial hindrance to arterial inflow, when applied to a subjective perceptual pressure of 7 out of 10, with 0 being no perceived pressure and 10 being maximal pressure. Using this information, we recruited four athletes prior to the start of the 7-week training program in an effort to standardize the degree of elastic wrap tightening to be used in the study. First, the 7.6 cm wide elastic knee wraps were applied to the proximal-most arm and thigh with just enough tightness to remain in place, equating to a perceived pressure of 0. The wrap was then slowly pulled until the participant reported a perceived pressure of 7 out of 10 and was then secured, in alignment with previous methods (Wilson et al., 2013). The difference between the end of the wrap prior to tightening (perceived pressure = 0), and following tightening (perceived pressure = 7), was then calculated for all four participants. Wrap overlap was then averaged resulting in 7.0 cm (2.75in) for the thigh, and 6.6 cm (2.60in) for the arm. We then concluded that 7.6 cm (3.0in) of wrap tightening relative to limb circumference for both the thighs and arms would yield a perceptive pressure near 7 out of 10 (within reason) for most of the athletes participating. Another potential limitation is that the participants in this study were highly resistance-trained, male, collegiate American football players with average arm circumferences across the four training groups of between 31.7 and 36.3 cm (12.5–14.3in), and average thigh circumferences between 55.6 and 61.1 cm (21.9–24.1in). Thus, the resultant changes in endocrine physiology may not be generalizable to other populations with different degrees of resistance training experience or non-athletes. Future research should aim to investigate the acute physiological, and longitudinal adaptations, following the various practical BFR application methods outlined by Ancieto and da Silva Leandro, specifically the individualized tightening of elastic wraps based upon subjective perceptions of pressure and wrap tightening relative to various percentages of measured limb circumferences (Ancieto and da Silva Leandro, 2022), improving the use of BFR in field-based settings with large cohorts of athletes where the use of expensive, yet more precise, equipment may not be logistically and/or financially feasible.

## Conclusion

In conclusion, the present results suggest that well-trained NCAA Division II American football athletes supplementing traditional heavy-load resistance exercise regimens with low-load practical BFR experience statistically significant amplifications in acute sTes during both AM and PM training times, and non-significant elevations in sCort post-exercise in PM. These acute exercise-induced alterations do not appear to have lasting effects on resting hormonal concentrations, as pre-exercise levels remained unchanged following the 7-week strength and conditioning program. However, following the conclusion of the 7-week strength and conditioning program (i.e., Test 2) training sessions elicited nearly no sCort response for any group in the PM cohort, suggesting that monitoring the sCort response to a given bout of resistance exercise over time may be a valuable indicator of positive training adaptations in athletic populations as individuals adapt to a given exercise stressor. This null sCort response post-program for all four groups in the PM supports the significant increases in back squat and bench press 1RM strength found in previously published performance data from the same cohort of NCAA Division II American football players (Luebbers et al., 2014), suggesting favorable post-exercise T/C ratios may be indicative of positive training adaptations when monitored longitudinally.

## Data Availability

The raw data supporting the conclusions of this article will be made available by the authors, without undue reservation.
